# Supplementary material for: Regularization by neural style transfer for MRI field-transfer reconstruction with limited data
Source: Front Artif Intell. 2025 Jun 18;8:1579251. doi: 10.3389/frai.2025.1579251 (PMC12213525; doi:10.3389/frai.2025.1579251)
Supplement: Supplementary file 1 [file Supplementary_file_1.docx]

Supplementary Material

## Supplementary Figures


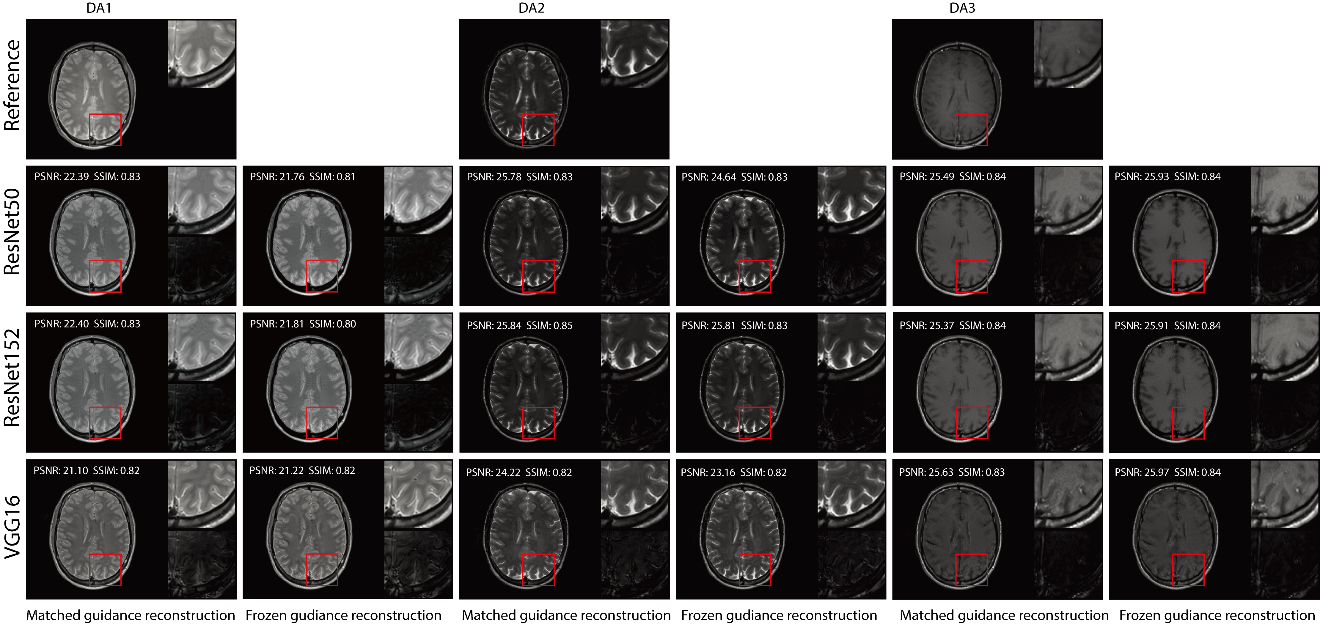


**Supplementary Figure S1.** Visual illustration of RNST results for matched and frozen guidance setups for three DAs on the original scans on the triple TSE dataset. The image details highlighted in the red box in each figure were enlarged on the upper right side, with the corresponding error maps compared to the registered reference showing on the lower right side.


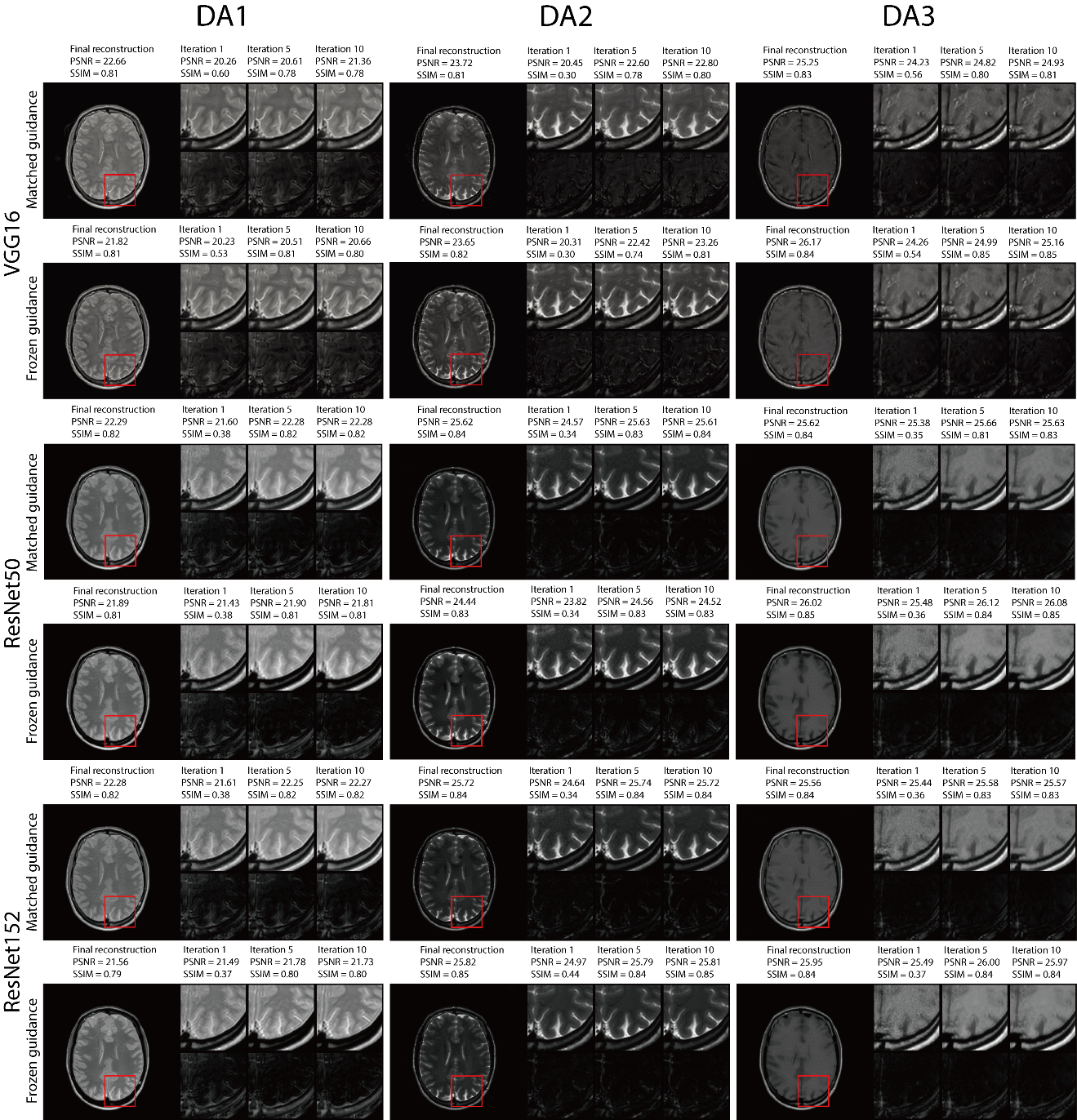


**Supplementary Figure S2.** Visual illustration and quantitative metrics of the RNST reconstructions across iterations with additional noise on the triple TSE dataset. We present intermediate steps for iterations 1, 5 and 10, together with their intermediate evaluation metrics and error maps.


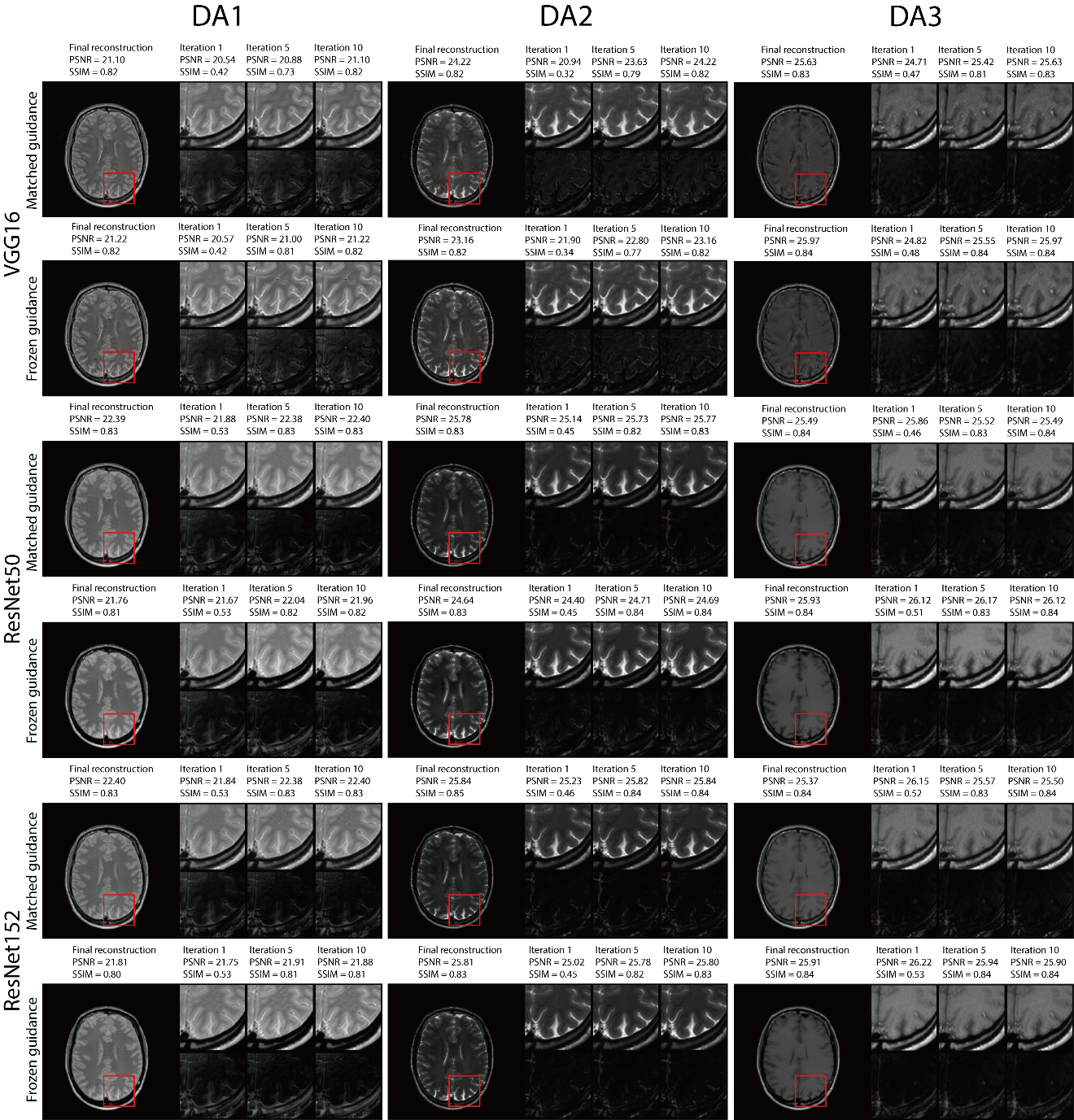


**Supplementary Figure S3.** Visual illustration and quantitative metrics of the RNST reconstructions across iterations on the original scans on the triple TSE dataset. We present intermediate steps for iterations 1, 5 and 10, together with their intermediate evaluation metrics and error maps.
